# Circulating apelin, chemerin and omentin levels in patients with gestational diabetes mellitus: a systematic review and meta-analysis

**DOI:** 10.1186/s12944-020-01209-7

**Published:** 2020-02-22

**Authors:** Jianran Sun, Jiale Ren, Chunlin Zuo, Datong Deng, Faming Pan, Ruoping Chen, Jie Zhu, Chao Chen, Shandong Ye

**Affiliations:** 1 Division of Life Science and Medicine, Department of Endocrinology, The First Affiliated Hospital of USTC (Anhui Provincial Hospital), University of Science and Technology of China, 17 Lujiang Road, Hefei, 230001 China; 2 Department of Rheumatology and Immunology, Arthritis Research Institute, The First Affiliated Hospital of Anhui Medical University, 218 Jixi Road, Hefei, 230022 Anhui China; 3 Department of Endocrinology, Institute of Endocrinology and Metabolism, The First Affiliated Hospital of Anhui Medical University, 218 Jixi Road, Hefei, 230022 Anhui China; 4 Department of Epidemiology and Biostatistics, School of Public Health, Anhui Medical University, 81Meishan Road, Hefei, 230032 Anhui China

**Keywords:** Apelin, Chemerin, Omentin, Gestational diabetes mellitus, Meta-analysis

## Abstract

**Background:**

The available data on the significance of circulating apelin, chemerin and omentin in women with gestational diabetes mellitus (GDM) are inconsistent. This analysis includes a systematic review of the evidence associating the serum concentrations of these adipokines with GDM.

**Methods:**

Publications through December 2019 were retrieved from PubMed, Embase, the Cochrane Library, and Web of Science. Subgroup analysis and meta-regression were conducted to evaluate sources of heterogeneity.

**Results:**

Analysis of 20 studies, including 1493 GDM patients and 1488 normal pregnant women did not find significant differences in circulating apelin and chemerin levels (apelin standardized mean difference [SMD] = 0.43, 95% confidence interval (CI): − 0.40 to 1.26, *P* = 0.31; chemerin SMD = 0.77, 95% CI − 0.07 to 1.61, *P* = 0.07). Circulating omentin was significantly lower in women with GDM than in healthy controls (SMD = − 0.72, 95% CI − 1.26 to − 0.19, *P* = 0.007). Publication bias was not found; sensitivity analysis confirmed the robustness of the pooled results.

**Conclusions:**

Circulating omentin was decreased in GDM patients, but apelin and chemerin levels were not changed. The results suggest that omentin has potential as a novel biomarker for the prediction and early diagnosis of GDM.

## Background

Gestational diabetes mellitus (GDM) affects 5 to 20% of pregnant women, depending on the diagnostic criteria, population, and racial or ethnic group. According to the American Diabetes Association (ADA) criteria, GDM is defined as diabetes diagnosed in the second or third trimester of pregnancy that was not overt prior to gestation [1, 2]. GDM increases the risk of hypoglycemia, hypocalcemia, and hyperbilirubinemia at birth, and the risk of developing glucose intolerance during childhood, adolescence, or adulthood [3, 4].

Understanding the pathogenesis of GDM is the key to preventing its development during pregnancy [5]. A progressive decline in insulin sensitivity and the development of β-cell dysfunction are believed to increase the risk of GDM [6, 7]. Recent evidence indicates that dysregulation of the secretion of adipokines, which are produced by adipose tissue, is involved in the development of insulin resistance (IR) during pregnancy [8].

Apelin, a bioactive peptide, was first identified in an extract of bovine stomach tissue. Both apelin and its receptor are expressed in various tissues, including the central nervous system, adipocytes, and the placenta [9]. Elsehmawy et al. [10] have described apelin as a novel adipokine that is produced and secreted by mature human adipocytes. Studies of serum or plasma apelin concentration in GDM patients by Baris et al. found lower circulating apelin in women with GDM than in controls; however, Emel et al. reported an increased apelin concentration in GDM patients [11, 12].

Chemerin, also called retinoic acid receptor responder protein 2 (RARRES2) or tazarotene-induced gene 2 protein (TIG2), is translated as an 18 kDa inactive precursor protein and converted to the 16 kDa active form by cleavage of the C-terminus by an extracellular serine protease [13]. Chemerin was identified as an adipokine by Bozaoglu et al. who described its activity in regulating adipogenesis and adipocyte metabolism associated with metabolic syndrome [14]. Not all studies found a correlation between circulating chemerin and GDM. Yang et al. found that circulating chemerin was significantly elevated in women with GDM compared to controls, while Sadia et al. failed to find a significant association between chemerin concentration and GDM [15, 16].

Omentin, or intelectin-1, is an adipokine consisting of 313 amino acids primarily secreted from visceral adipose tissue, but it is also expressed in the heart, placenta, and ovaries [17]. Adipokines likely influence GDM; however, data on the roles of apelin, chemerin, and omentin in GDM are limited and inconsistent [18]. The aim of this study was to systematically evaluate the correlations between the serum levels of such adipokines and GDM.

## Materials and methods

### Search strategy

The meta-analysis was performed following the Preferred Reporting Items for Systematic Reviews and Meta-analysis (PRISMA) guidelines [19]. Studies published in English between January 1970 and December 2019 were retrieved from PubMed, Embase, the Cochrane Library, and the Web of Science using the text and MeSH terms “adipokines” and “gestational diabetes mellitus.” The search terms included “apelin” or “apelin, AGTRL1 ligand, human” or “chemerin” or “chemerin proteins” or “omentin” or “intelectin, human” combined with “gestational diabetes mellitus” or “GDM”. A full description of the search terms and strategy is available in the Supplementary material.

### Inclusion and exclusion criteria

The inclusion criteria were as follow: a) Studies of any design, including cross-sectional, case-control, and clinical cohort studies; b) studies providing detailed data regarding serum or plasma apelin, chemerin, or omentin levels in patients with GDM and healthy controls; and c) studies in which the language was restricted to English.

The exclusion criteria were as follows: a) Studies that evaluated women with all types of diabetes mellitus (e.g., type 1 diabetes, type 2 diabetes, or specific types of diabetes due to other causes) and did not provide precise data on GDM; b) studies of fetal and/or placental tissues, such as cord blood an placental biopsies; c) studies utilizing animal models, cell cultures (in vitro or ex vivo), tissue-based cultures, and mRNA expression; d) and conference abstracts, case reports, editorials, comments or review articles, and articles that lack original data.

### Quality assessment of studies and data extraction

The quality of observational studies was evaluated using the Newcastle-Ottawa quality assessment scale (NOS) described by Wells et al. [20]. The NOS tool includes nine items with scores ranging from 0 to 9. A higher score indicates a higher quality. Two investigators (JS and JR) independently reviewed the retrieved studies, and extracted the data to a table for review by a third investigator (CZ). Disagreements between investigators were resolved by discussion among all investigators. The first author’s name, study sample size, year of publication, geographic region, study design, ethnicity, trimester in which the adipokine assays were performed, age, body mass index (BMI) and adipokine concentration in GDM patients (all means ± standard deviation) were collected.

### Statistical analysis

Statistical analysis was performed using STATA version 12.0 (Stata Corporation, College Station, TX, USA). The study effect sizes were described by SMDs and 95% CIs. The results were reported as means and SD in most studies. A few studies reported results as medians with maxima and minima or medians and interquartile range. For those studies, the raw data were transformed to estimate the mean and SD. The method and its accuracy have been described elsewhere [21, 22]. Cochrane’s *Q* (chi square test) and the *I*^*2*^ metric (*I*^*2*^ = [(*Q*-df) / *Q*) × 100%] were used to assess heterogeneity. *I*^*2*^ values of 25, 50, and 75% indicated low, medium, and high heterogeneity, respectively. A random-effect model (the DerSimonian and Laird method) was used in cases of heterogeneity, otherwise a fixed-effect model (the Mantel-Haenszel method) was used. Subgroup analysis and meta-regression analysis were used to determine the source of heterogeneity. Funnel plots, Begg’s test and Egger’s test were used to detect publication bias, with sensitivity analysis to identify the outliers if there was high heterogeneity. *P*-values < 0.05 were considered statistically significant.

## Results

### Literature research and characteristics of the included studies

**Fig. 1:**
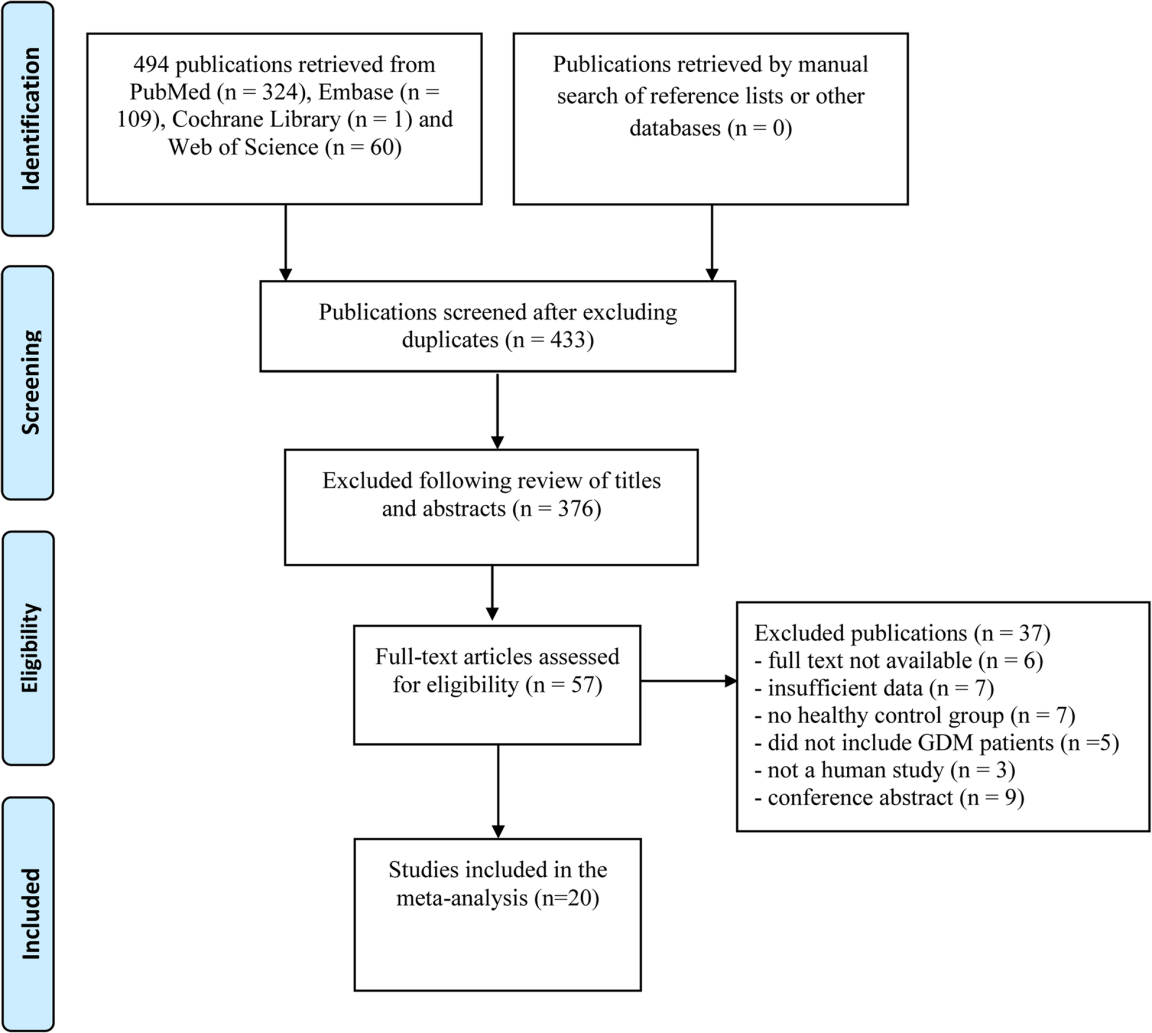
Flow chart for the selection of included studies

Of the 494 articles that were retrieved, 20 were included in the meta-analysis; four were apelin studies [4, 6, 7, 11], nine were chemerin studies [5, 8, 15, 16, 23–27], five were omentin studies [28–32], one study involved both apelin and omentin [12], and one study involved both chemerin and omentin [33]. The 20 studies enrolled 2981 participants, of which 1493 were GDM patients and 1488 were healthy pregnant women (controls) [4–8, 11, 12, 15, 16, 23–33] (Fig. 1). The five apelin studies included 336 GDM patients and 237 controls. The 10 chemerin studies included 772 GDM patients and 857 controls. The five omentin studies included 385 GDM patients and 394 controls.

The 20 articles were published from 2010 to 2019. GDM was diagnosed based on the ADA criteria in five studies, the American College of Obstetricians and Gynecologists (ACOG) criteria in three studies, the Carpenter and Couston (C&C) criteria in two studies, the World Health Organization (WHO) criteria in three studies, the International Association of Diabetes and Pregnancy Study Group (IADPSG) criteria in four studies, the Australasian Diabetes in Pregnancy Society (ADIPS) criteria in two studies, and National Diabetes Date Group (NDDG) criteria in one study. Apelin, chemerin, and omentin were measured by enzyme linked immunosorbent assay (ELISA) in 18 studies and by radioimmunoassay in two studies. The NOS scores ranged from 6 to 8. The characteristics of the 20 included studies are shown in Table 1.

**Table 1:** Characteristics of studies investigating circulating apelin, chemerin and omentin levels in GDM

					Cases				Controls						
First authors	Year	Country	Study type	N	Age (years), mean ± SD	Gestational BMI(kg/m^2^)	Mean ± SD(ng/mL)/(ng/mL)	N	Age (years), mean ± SD	Gestational BMI(kg/m^2^)	Mean ± SD(ng/mL)/(ng/mL)	*P*	Criteria for GDM	Measurement	NOS
Apelin															
Baris [11]	2014	Turkey	Cohort	141	35.18 ± 5.55	26.82 ± 4.25	0.33 ± 0.18	49	34.89 ± 5.64	26.50 ± 2.66	0.56 ± 0.29	< 0.001	ADA	ELISA system)	7
Mehmet [6]	2012	Turkey	CC	30	30.90 ± 4.20	25.90 ± 3.30	13.50 ± 9.30	30	31.00 ± 3.20	25.70 ± 2.80	9.60 ± 5.90	0.001	ACOG	ELISA	8
Emel [12]	2016	Turkey	Cohort	20	28.95 ± 6.21	27.95 ± 4.19	2.29 ± 0.39	20	27.00 ± 1.20	27.05 ± 4.05	0.91 ± 0.32	< 0.001	ACOG	ELISA	6
Oncul [7]	2013	Turkey	CC	24	34.00 ± 7.00	31.32 ± 5.82	0.15 ± 0.05	21	30.00 ± 4.00	29.32 ± 4.53	0.16 ± 0.09	0.602	C&C	ELISA	7
Beata [4]	2010	Poland	CC	101	31.00 ± 6.02	24.57 ± 5.11	1.65 ± 0.39	101	30.00 ± 4.51	23.39 ± 4.21	1.65 ± 0.32	> 0.05	WHO	RIA	7
Beata [4]	2010	Poland	CC	20	31.65 ± 3.76	20.66 ± 4.51	1.61 ± 0.24	16	29.65 ± 5.26	25.94 ± 4.51	1.59 ± 0.49	> 0.05	WHO	RIA	7
Chemerin															
Panayoula [33]	2018	Greece	CC	5	29.80 ± 1.20	26.90 ± 0.80	209.39 ± 37.60	13	30.60 ± 3.40	26.90 ± 0.60	162.27 ± 10.04	> 0.05	IADPSG	ELISA	7
Panayoula [33]	2018 Greece	Greece	CC	10	27.70 ± 1.60	36.00 ± 1.50	215.16 ± 17.19	10	36.10 ± 1.20	36.40 ± 1.80	212.81 ± 36.22	> 0.05	IADPSG	ELISA	7
Gillian [23]	2012	Australia	CC	69	35.20 ± 0.60	31.20 ± 0.90	117.60 ± 3.50	62	33.10 ± 0.60	34.80 ± 1.00	124.20 ± 4.00	> 0.05	ADIPS	ELISA	7
Boyadzhieva [5]	2013	Bulgarias	CC	127	32.20 ± 5.20	28.50 ± 6.60	6.89 ± 2.26	109	30.60 ± 4.40	28.00 ± 5.40	7.97 ± 2.42	0.009	IADPSG	ELISA	8
Syeda [26]	2017	Pakistan	CC	208	27.30 ± 5.56	24.83 ± 5.13	93.39 ± 45.43	300	25.78 ± 4.73	22.38 ± 3.93	14.35 ± 5.88	< 0.001	IADPSG	ELISA	8
Umit [27]	2016	Turkey	CS	76	27.59 ± 12.09	36.25 ± 22.21	4.53 ± 3.82	82	26.35 ± 12.833	31.10 ± 21.05	3.43 ± 1.84	0.100	ACOG	ELISA	6
Li [24]	2015	China	CC	16	29.30 ± 2.50	23.00 ± 0.40	222.00 ± 25.20	15	28.30 ± 2.40	20.90 ± 0.50	73.10 ± 8.60	< 0.05	ADA	ELISA	7
Li [24]	2015	China	CC	16	29.30 ± 3.10	27.10 ± 0.40	225.20 ± 26.80	15	29.40 ± 2.80	26.90 ± 0.30	151.00 ± 15.50	< 0.05	ADA	ELISA	7
Li [24]	2015	China	CC	16	29.14 ± 4.38	33.00 ± 0.80	136.80 ± 20.30	12	29.50 ± 4.84	33.40 ± 0.80	195.00 ± 34.40	> 0.05	ADA	ELISA	7
Liang [25]	2018	China	CC	46	31.89 ± 4.53	21.52 ± 3.38	20.11 ± 3.28	43	30.58 ± 3.77	21.06 ± 2.93	17.63 ± 3.63	0.001	ADA	ELISA	8
Sadia [16]	2018	Canada	Cohort	105	35.00 ± 4.00	25.78 ± 5.19	54.80 ± 16.00	76	35.00 ± 4.00	24.79 ± 5.21	55.80 ± 12.70	0.48	NDDG	ELISA	8
Dorte [8]	2010	Germany	CC	40	33.00 ± 10.00	24.90 ± 4.90	217.60 ± 72.30	80	28.00 ± 5.00	22.30 ± 7.00	230.30 ± 42.40	> 0.05	ADA	ELISA	8
Yang [15]	2017	China	CC	19	26.84 ± 2.95	22.74 ± 1.68	146.60 ± 38.91	20	26.84 ± 2.95	21.72 ± 1.23	187.23 ± 46.83	< 0.05	IADPSG	ELISA	7
Yang [15]	2017	China	CC	19	26.84 ± 2.95	25.67 ± 1.39	308.56 ± 56.43	20	26.84 ± 2.95	24.15 ± 1.24	227.53 ± 46.49	< 0.05	IADPSG	ELISA	7
Omentin															
Panayoula [33]	2018	Greece	CC	5	29.80 ± 1.20	26.90 ± 0.80	17.69 ± 2.46	13	30.60 ± 3.40	26.90 ± 0.60	31.85 ± 6.67	< 0.05	IADPSG	ELISA	7
Panayoula [33]	2018	Greece	CC	10	27.70 ± 1.60	36.00 ± 1.50	19.20 ± 1.60	10	36.10 ± 1.20	36.40 ± 1.80	20.25 ± 3.55	< 0.05	IADPSG	ELISA	7
Aktas [28]	2014	Turkey	Cohort	36	28.60 ± 7.20	28.40 ± 13.74	304.27 ± 413.06	37	48.00 ± 8.60	27.00 ± 12.34	363.28 ± 488.19	0.001	C&C	ELISA	8
Gillian [29]	2012	Australia	Cohort	21	35.50 ± 0.80	23.20 ± 0.80	12.10 ± 1.40	27	33.10 ± 0.70	23.40 ± 0.60	19.50 ± 2.30	< 0.05	ADIPS	ELISA	8
Gillian [29]	2012	Australia	Cohort	18	34.00 ± 1.10	37.80 ± 1.50	8.20 ± 1.20	17	30.80 ± 1.10	38.60 ± 1.30	7.10 ± 0.90	> 0.05	ADIPS	ELISA	8
Emel [12]	2016	Turkey	Cohort	20	28.95 ± 6.21	27.95 ± 4.19	13.11 ± 1.79	20	27.00 ± 1.20	27.05 ± 4.05	18.64 ± 3.48	< 0.001	ACOG	ELISA	6
Marie [31]	2018	Australia	Cohort	96	34.10 ± 7.40	28.00 ± 6.60	157.00 ± 83.00	96	32.80 ± 9.30	26.30 ± 4.70	158.00 ± 93.00	0.94	ADA	ELISA	8
Marie [31]	2018	Australia	Cohort	96	34.10 ± 7.40	28.00 ± 6.60	118.00 ± 77.00	96	32.80 ± 9.30	26.30 ± 4.70	150.00 ± 89.00	0.12	ADA	ELISA	8
Lewandowski [30]	2010	Poland	CC	20	29.70 ± 5.50	28.10 ± 4.70	48.00 ± 12.00	23	28.30 ± 3.90	28.30 ± 4.80	50.20 ± 7.90	0.64	WHO	ELISA	6
RadzisBaw [32]	2018	Poland	CC	63	12.00 ± 3.87	9.43 ± 1.67	438.36 ± 373.28	55	11.09 ± 3.43	8.18 ± 1.18	528.40 ± 374.55	> 0.05	WHO	ELISA	7

*N* Number of studies, *GDM* Gestational diabetes mellitus, *CC* Case control, *BMI* Body mass index, *ADA* American diabetes association, *ACOG* American College of Obstetricians and Gynecologists;

*C&C* Carpenter and Couston, *WHO* World Health Organization, *IADPSG* International Association of Diabetes and Pregnancy Study Group, *ADIPS* Australasian Diabetes in Pregnancy Society

*NDDG* National Diabetes Date Group, *ELASA* Enzyme linked immunosorbent assay, *RIA* Radioimmunoassay, *NOS* Newcastle-Ottawa Scale

### Overall effects

The results showed that there was no significant difference between GDM patients and normal controls in circulating apelin (SMD = 0.43, 95% CI − 0.40 to 1.26, *P* = 0.31) and chemerin (SMD = 0.77, 95% CI − 0.07 to 1.61, *P* = 0.07) levels. Circulating omentin was significantly lower in women with GDM than in healthy controls (SMD = − 0.72, 95% CI − 1.26 to − 0.19, *P* = 0.007).

### Heterogeneity

**Fig. 2:**
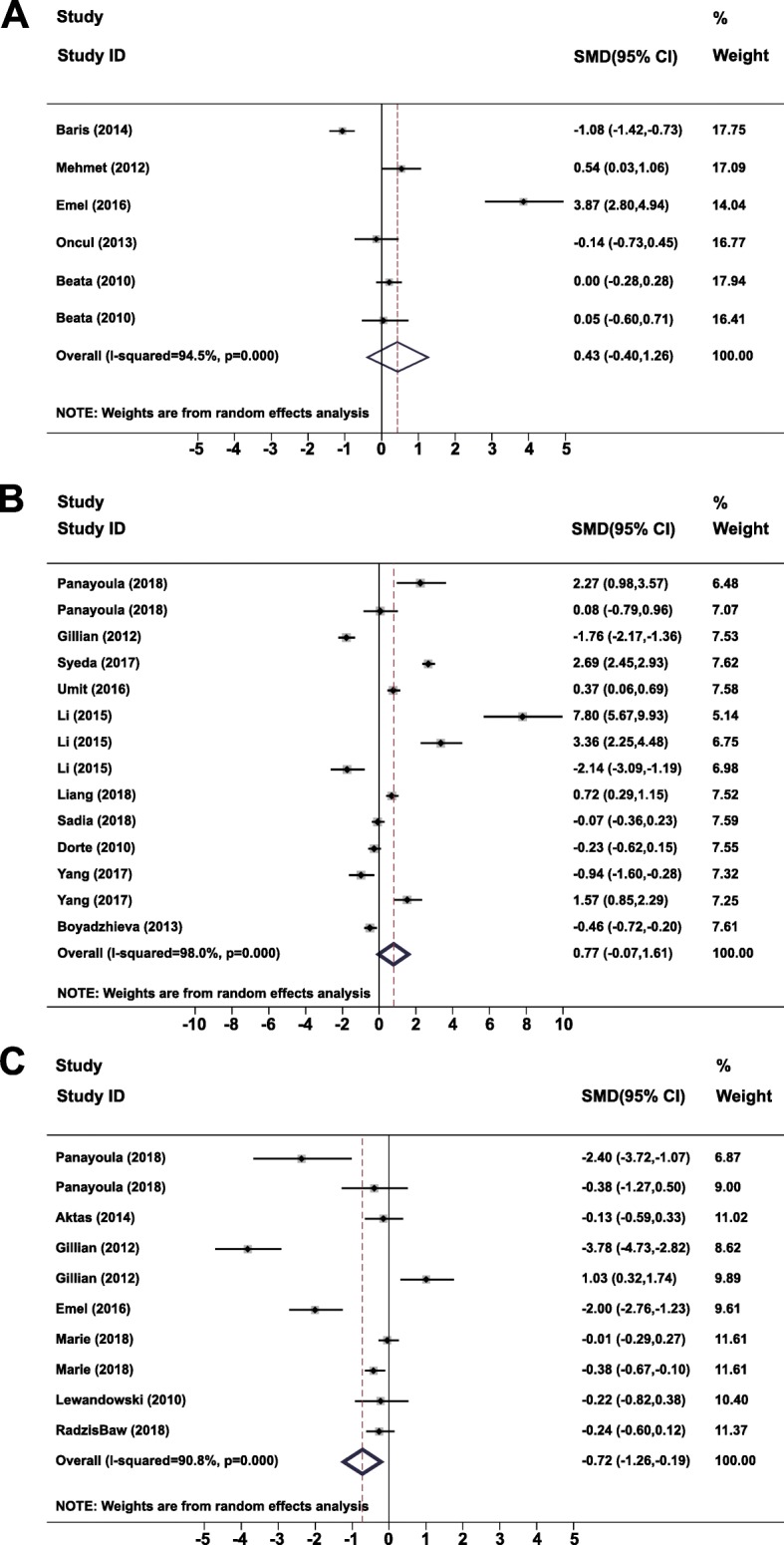
Forest plots of heterogeneity in studies comparing serum (**a**) apelin (**b**) chemerin, and (**c**) omentin in GDM patients versus healthy controls

Significant heterogeneity was detected in the apelin (*I*^*2*^ = 94.5%, *P* < 0.001), chemerin (*I*^*2*^ = 98.0%, *P* < 0.001), and omentin (*I*^*2*^ = 90.8%, *P* < 0.001) studies. The random-effect model was used for the meta-analysis (Fig. 2).

### Subgroup analysis

The results of subgroup analyses of the study results stratified by ethnicity, age, BMI, study type, measurement type, diagnostic criteria, and trimester that the adipokine measurements were performed are shown in Table 2. Apelin levels were lower in women with GDM diagnosed by the ADA criteria than in controls, but the difference was significant with some but not all the commercially available ELISA kits. Asian and African women, patients younger than 30 years of age, those with BMIs ≥28 kg/m^2^, and those diagnosed using the ACOG criteria in the second trimester had higher circulating chemerin levels than controls. Chemerin levels in GDM patients and controls did not differ in the other subgroups. Omentin was lower in GDM patients than in controls in Caucasian women, patients younger than 30 years of age, those with a BMI < 28 kg/m^2^, or those diagnosed in the second trimester of pregnancy. No differences in the other subgroups were significant. Subgroup analysis suggested that differences in the ELISA kits used in the various studies may be the primary source of heterogeneity in the reported serum apelin levels.

**Table 2:** Stratified meta-analysis of circulating apelin, chemerin and omentin levels in GDM

Subgroups	N	Test of association			Test of heterogeneity		
		SMD (95% CI)	z	*P*	*Q*	*I*^*2*^*(%)*	*P*
Apelin							
Ethnicity							
Caucasian	2	0.01 (− 0.40 to 1.26)	0.06	0.95	0.02	0.00	0.88
Asian	4	0.72 (− 0.77 to 2.22)	0.95	0.35	88.68	96.60	< 0.05
Combined	6	0.43 (−0.40 to 1.26)	1.02	0.31	90.85	94.50	< 0.05
Age(mean,years) yyeyeyears)							
< 30	2	2.17 (−1.09 to 5.43)	1.31	0.19	30.23	88.20	< 0.05
≥ 30	4	−0.31 (− 0.93 to 0.31)	0.99	0.32	25.41	96.70	< 0.05
Combined	6	0.43 (− 0.40 to 1.26)	1.02	0.31	90.85	94.50	< 0.05
BMI(mean,kg/m^2^)							
< 28	4	−0.14 (− 0.85 to 0.57)	0.39	0.70	35.40	91.50	< 0.05
≥ 28	2	1.84 (−2.09 to 5.77)	0.92	0.36	41.58	97.60	< 0.05
Combined	6	0.43 (−0.40 to 1.26)	1.02	0.31	90.85	94.50	< 0.05
Study type							
Case-control	4	0.10 (−0.17 to 0.36)	0.72	0.48	3.95	24.00	0.27
Cohort	2	1.37 (−3.48 to 6.22)	0.55	0.58	74.68	98.70	< 0.05
Combined	6	0.43 (−0.40 to 1.26)	1.02	0.31	90.85	94.50	< 0.05
Measurement type							
ELISA	4	0.72 (−0.77 to 2.22)	0.95	0.35	88.68	96.60	< 0.05
RIA	2	0.01 (−0.25 to 0.26)	0.06	0.95	0.02	0.00	0.88
Combined	6	0.43 (−0.40 to 1.26)	1.02	0.31	90.85	94.50	< 0.05
ELISA kits							
Phoenix Pharmaceuticals	**1**	**−1.08 (−1.42 to − 0.73)**	**6.16**	**< 0.01**	0.02	0.00	0.23
Eastbiopharm	**1**	**0.54 (0.03 to 1.06)**	**2.06**	**0.04**	25.41	25.00	0.87
Bio-Tek Instruments	**1**	**3.87 (2.80 to 4.94)**	**7.10**	**< 0.01**	53.60	36.00	0.45
RayBiotech	1	−0.14 (− 0.72 to 0.45)	0.47	0.64	37.60	28.00	0.34
Combined	4	0.72 (−0.77 to 2.22)	0.95	0.34	88.68	96.60	< 0.05
Diagnostic criteria							
ADA	**1**	**−1.07 (−1.42 to − 0.73)**	**6.16**	**< 0.05**	0.00	NA	NA
ACOG	2	2.17 (−1.09 to 5.43)	1.31	0.19	30.23	96.70	< 0.05
C&C	1	−0.14 (− 0.73 to 0.45)	0.47	0.64	0.00	NA	NA
WHO	2	0.01 (−0.25 to 0.26)	0.06	0.95	0.02	0.00	0.88
Combined	6	0.43 (−0.40 to 1.26)	1.02	0.31	90.85	94.50	< 0.05
Measurement trimester							
Second	1	0.00 (−0.28 to 0.28)	0.00	1.00	89.44	95.50	< 0.05
Third	5	0.57 (−0.62 to 1.76)	0.94	0.35	0.00	NA	NA
Combined	6	0.43 (−0.40 to 1.26)	1.02	0.31	90.85	94.50	< 0.05
Chemerin							
Ethnicity							
Caucasian	5	−0.00 (− 0.44 to 0.43)	0.01	0.99	19.03	79.00	< 0.05
Asian	**7**	**1.21 (0.09 to 2.35)**	**2.11**	**0.04**	128.50	95.30	< 0.05
Australoid	1	−1.76 (−2.17 to 1.36)	8.53	0.36	0.00	NA	NA
African	**1**	**2.69 (2.45 to 2.93)**	**21.74**	**< 0.05**	0.00	NA	NA
Combined	14	0.77 (−0.07 to 1.61)	1.80	0.07	651.01	98.00	< 0.05
Age(mean,years)							
< 30	**9**	**1.51 (0.28 to 2.73)**	**2.41**	**0.02**	306.13	97.40	< 0.05
≥ 30	5	−0.36 (−1.03 to 0.30)	1.07	0.28	75.12	94.70	< 0.05
Combined	14	0.77 (−0.07 to 1.61)	1.80	0.07	651.01	98.00	< 0.05
BMI(mean,kg/m^2^)							
< 28	5	−0.75 (−1.62 to 0.11)	1.71	0.09	80.05	95.00	< 0.05
≥ 28	**9**	**1.69 (0.58 to 2.79)**	**2.99**	**< 0.05**	369.82	97.80	< 0.05
Combined	14	0.77 (−0.07 to 1.61)	1.80	0.07	651.01	98.00	< 0.05
Study type							
Case-control	13	0.86 (−0.08 to 1.80)	1.79	0.07	636.04	98.10	< 0.05
Cohort	1	−0.07 (− 0.36 to 0.23)	0.45	0.65	0.00	NA	NA
Combined	14	0.77 (−0.07 to 1.61)	1.80	0.07	651.01	98.00	< 0.05
ELISA kits							
Millipore	3	0.60 (− 0.50 to 1.71)	1.07	0.29	11.90	83.20	0.29
R&D systems	4	−0.11 (−1.64 to 1.41)	0.15	0.88	100.69	97.00	0.88
Sbjbio	3	2.94 (−2.25 to 0.12)	1.11	0.27	98.62	98.00	0.27
Biovendor	2	0.08 (−0.52 to 0.67)	0.26	0.80	5.78	82.70	0.80
other kits	2	1.11 (−1.97 to 4.20)	0.71	0.48	302.71	99.70	0.48
Combined	14	0.77 (−0.07 to 1.61)	1.80	0.07	651.01	98.00	0.07
Diagnostic criteria							
IADPSG	6	0.86 (− 0.72 to 2.43)	1.07	0.29	350.75	98.60	< 0.05
ADIPS	1	1.26 (−2.17 to 1.36)	8.53	> 0.05	0.00	NA	NA
ACOG	**1**	**0.37 (0.06 to 0.69)**	**2.31**	**0.02**	0.00	NA	NA
ADA	4	2.27 (−0.46 to 4.99)	1.63	0.10	98.95	97.00	< 0.05
NDDG	2	−0.13 (− 0.36 to 0.10)	1.10	0.27	0.46	0.00	0.49
Combined	14	0.77 (−0.07 to 1.61)	1.80	0.07	651.01	98.00	< 0.05
Measurement trimester							
Second	**8**	**1.09 (0.17 to 2.34)**	**1.70**	**0.04**	484.85	98.60	< 0.05
Third	6	0.40 (−0.61 to 1.41).	0.77	0.44	112.32	95.50	< 0.05
Combined	14	0.77 (−0.07 to 1.61)	1.80	0.07	651.01	98.00	< 0.05
Omentin							
Ethnicity							
Caucasian	**6**	**−0.33 (−0.65 to − 0.02)**	**2.06**	**0.04**	13.78	63.70	0.02
Asian	2	−1.04 (−2.87 to 0.79)	1.11	0.27	16.82	94.10	< 0.05
Australoid	2	− 1.36 (−6.07 to 3.35)	0.57	0.57	62.46	98.40	< 0.05
Combined	**10**	**−0.73 (−1.26 to − 0.20)**	**2.68**	**< 0.05**	97.49	90.80	< 0.05
Age(mean,years)							
< 30	**6**	**−0.75 (−1.36 to − 0.15)**	**2.44**	**0.02**	27.75	82.00	< 0.05
≥ 30	4	−0.69 (−1.73 to 0.34)	1.31	0.19	68.06	95.60	< 0.05
Combined	**10**	**−0.73 (−1.26 to − 0.20)**	**2.68**	**< 0.05**	97.49	90.80	< 0.05
BMI(mean,kg/m^2^)							
< 28	**4**	**−2.06 (−3.76 to − 0.37)**	**2.38**	**0.02**	59.74	95.00	< 0.05
≥ 28	6	−0.06 (− 0.38 to 0.27)	0.35	0.73	14.48	65.50	0.01
Combined	**10**	**−0.73 (−1.26 to − 0.20)**	**2.68**	**< 0.05**	97.49	90.80	< 0.05
Study type							
Case-control	4	−0.57 (−1.20 to 0.05)	1.79	0.07	9.72	69.10	0.02
Cohort	6	−0.79 (−1.58 to 0.10)	1.96	0.05	87.74	94.30	< 0.05
Combined	10	−0.73 (−1.26 to 0.20)	2.68	> 0.05	97.49	90.80	< 0.05
Diagnostic criteria							
IADPSG	2	−1.33 (−3.30 to 0.65)	1.32	0.19	6.15	83.80	0.01
C&C	2	−1.04 (−2.87 to 0.79)	1.11	0.27	16.82	94.10	< 0.05
ADIPS	2	−1.36 (−6.07 to 3.35)	0.57	0.57	62.46	98.40	< 0.05
ADA	2	−0.20 (− 0.56 to 0.17)	1.06	0.29	3.31	69.80	0.07
WHO	2	−0.24 (− 0.55 to 0.08)	1.48	0.14	0.00	0.00	0.95
Combined	**10**	**−0.73 (−1.26 to − 0.20)**	**2.68**	**< 0.05**	97.49	90.80	< 0.05
ELISA kits							
Millipore	2	−1.33 (−3.29 to 0.65)	1.32	0.18	6.15	83.80	0.01
Cusabio	2	−1.36 (−6.07 to 3.35)	0.57	0.57	62.46	98.40	< 0.05
Life Science	2	−0.19 (−0.56 to 0.17)	1.06	0.29	3.31	69.80	0.06
Bio Vendor	2	−1.09 (−2.81 to 0.64)	1.24	0.21	16.53	94.00	< 0.05
other kits	2	−0.16 (−0.53 to 0.20)	0.88	0.38	0.05	0.00	0.82
Combined	**10**	**−0.73 (−1.26 to − 0.19)**	**2.68**	**< 0.05**	97.49	90.80	< 0.05
Measurement trimester							
Second	**4**	**−0.12 (−0.31 to − 0.08)**	**1.19**	**0.04**	84.73	94.10	< 0.05
Third	6	−1.27 (−2.45 to 0.08)	2.10	0.23	1.10	0.00	0.78
Combined	**10**	**−0.73 (−1.26 to − 0.20)**	**2.68**	**< 0.05**	97.49	90.80	< 0.05

*N* Number of studies, *SMD* Standardized mean difference, *BMI* Body mass index, *ELASA* Enzyme linked immunosorbent assay, *RIA* Radioimmunoassay, *NA* Not available

### Meta-regression

Meta-regression analysis was used to further identify sources of heterogeneity for chemerin and omentin (Table 3). Explanatory covariates included publication year, geographic region, sample size, BMI, and gestational age. However, none of those covariates changed the correlation between serum chemerin or omentin levels and GDM risk.

**Table 3:** Univariate meta-regression analysis of heterogeneity caused by patient variables across studies

Variables	Coefficient	Standard error	95% CI	*t*	*P*
Chemerin					
Publication year	0.19	0.27	[− 0.40, 0.79]	0.71	0.50
Geographic region	0.37	3.18	[−7.40, 8.15]	0.12	0.91
Sample size	0.98	0.96	[−1.11, 3.07]	1.02	0.33
BMI	6.39	3.80	[−1.89, 14.68]	1.68	0.12
Gestational age	8.26	6.89	[−6.77, 23.29]	1.20	0.25
Omentin					
Publication year	−18.92	317.55	[−751.20, 713.36]	−0.06	0.95
Geographic region	−1.05	1.22	[−4.19, 2.09]	−0.86	0.43
Sample size	−1.33	0.70	[−2.93, 0.28]	−1.91	0.09
BMI	−2.10	1.74	[−6.11, 1.90]	−1.21	0.26
Gestational age	0.17	2.13	[−4.74, 5.09]	0.08	0.94

### Publication bias and sensitivity analysis

**Fig. 3:**
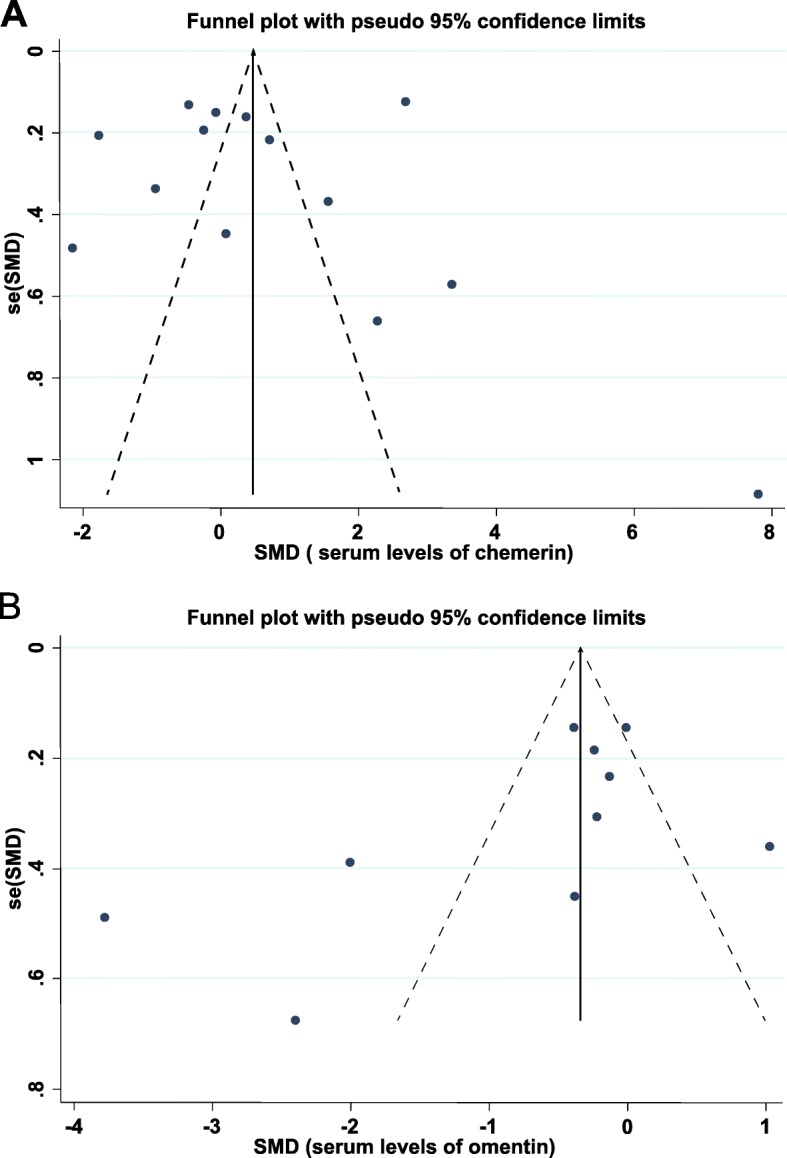
Funnel plots for estimating publication bias in studies of (**a**) chemerin and (**b**) omentin in women with GDM

**Fig. 4:**
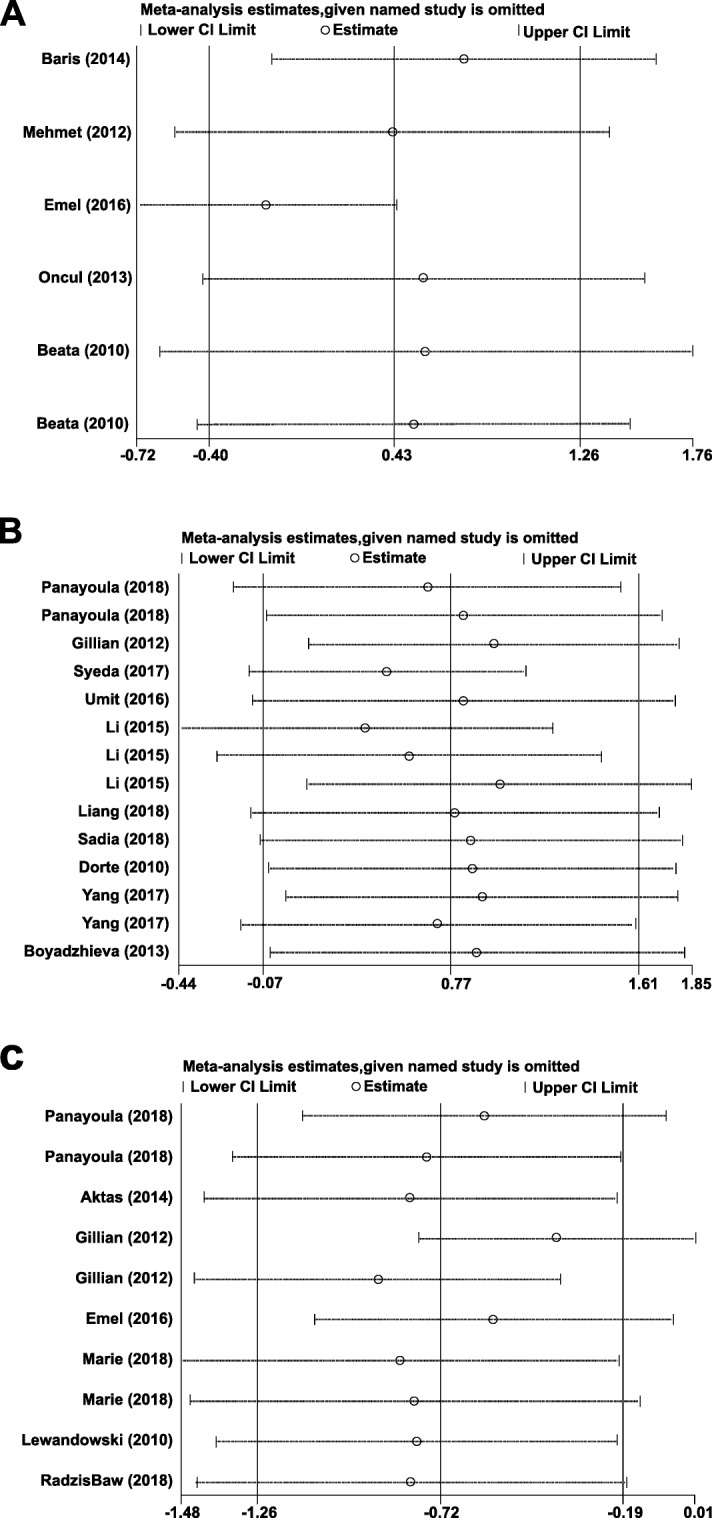
Sensitivity analysis plots of differences in serum (**a**) apelin (**b**) chemerin, and (**c**) omentin levels in GDM patients and healthy controls

Funnel plot symmetry indicated no significant publication bias among the studies (Fig. 3 and Table 4). The removal of any individual study in the sensitivity analysis did not change the overall statistical significance, indicating that that the results were statistically robust and reliable (Fig. 4).

**Table 4:** Heterogeneity and publication bias in studies of serum apelin, chemerin and omentin in women with GDM

			Heterogeneity test			Egger’s test		Begg’s test
Adipokine in GDM	N	SMD (95% CI)	*I*^*2*^	*P*	*t*	*P*	*z*	*P*
Apelin in GDM	6	0.43 (−0.40 to 1.26)	94.5	< 0.001	1.66	0.17	0.94	0.35
Chemerin in GDM	14	0.77 (−0.07 to 1.61)	98.0	< 0.001	0.03	0.98	1.26	0.21
Omentin in GDM	10	−0.72 (−1.26 to − 0.19)	90.8	< 0.001	−1.73	0.12	−1.70	0.09

*N* Number of studies, *SMD* Standardized mean difference

## Discussion

The meta-analysis of the association of circulating apelin, chemerin, and omentin with GDM included 20 studies, and found that apelin and chemerin levels in women with GDM did not differ significantly from those of controls with normal glucose tolerance. Omentin levels were lower in GDM patients than in women with normal pregnancies. Sensitivity analysis indicated that the pooled results were stable when the data of each individual study were deleted in succession. We did not find significant publication bias.

Apelin and its G protein-coupled receptor (APJ, apelin-angiotensin receptor-like 1) regulate glucose homeostasis in humans by increasing both glucose uptake and insulin sensitivity [34]. The increase of apelin levels in type 2 diabetes mellitus (T2DM) or obesity indicates an association between apelinemia and impaired glucose regulation [35]. Apelin stimulates various signaling pathways that decrease both cAMP and cAMP-induced AMPK activation. AMPK is a regulator of intracellular energy metabolism [36]. Apelin has been shown to decrease glucose transport in enterocytes by inhibiting sodium-dependent glucose transporter (SGLT)-1 [37]. In this study, apelin may have indirectly promoted an increase in plasma glucose by inhibiting glucose transport.

Apelin is present in human breast milk and may promote growth and regulate energy production in lactating infants [38]. In a recent review of clinical studies investigating the relationship between apelin and GDM, nearly all of the studies showed that lower apelin levels occurred in women with GDM compared to those without GDM. However, a few studies reported that the level of apelin-36 was increased in GDM [18].

Consistent with studies by Oncul et al. and Beata et al., we found that there were no significant differences in circulating apelin levels between GDM patients and controls with normal glucose tolerance [7]. In women diagnosed with GDM following the ADA criteria, circulating apelin was lower than that in controls, indicating that the diagnostic criteria may have affected the results obtained in previous studies. In addition, our subgroup analysis found significant heterogeneity in serum apelin levels in GDM that was associated with ELISA kits. Differences in the sensitivity and detection limits of the various ELISA kits resulted in heterogenous results. We found that the year of publication, geographic region, study sample size, BMI, and gestational age were not sources of heterogeneity across the included studies.

The binding of chemerin to its receptors can promote the recruitment of macrophages and dendritic cells, and neutrophil activation to produce inflammation [39], and chronic low-grade inflammation has been associated with increased IR [40]. A reduction in chemerin levels may be associated with the development of GDM through decreased insulin sensitivity and attenuated anti-inflammatory capacity [25].

The finding in this meta-analysis that circulating chemerin levels were similar in women with GDM and in normal pregnant women is consistent with previous studies [8, 23]. Unlike Zhou et al., this meta-analysis took ethnicity, diagnostic criteria, and sample size into account [41]. Sub-analysis revealed that Asian and African women, patients younger than 30 years of age, those with BMIs ≥28 kg/m^2^, or those diagnosed using the ACOG criteria in the second trimester had higher circulating chemerin levels than controls. Firstly, women with GDM and high BMIs had significantly higher chemerin concentrations than normal-weight women with GDM, consistent with a positive correlation of chemerin level and BMI. Obesity may have caused an increase in chemerin in pregnant women before delivery [15]. The results are consistent with those of Van Poppel et al., who found that circulating chemerin in pregnancy was influenced by maternal obesity status rather than GDM [42].

Secondly, the subgroup analysis also found that GDM patients younger than 30 years of age had a mean chemerin concentration 1.51 times that of normal controls, which indicates a negative correlation of age and chemerin concentration in GDM. Thirdly, chemerin levels were higher in GDM diagnosed during the second trimester than in controls. This result differs from that of Yang et al. who reported higher circulating chemerin in the third trimester than in early pregnancy [15]. A recent study found that both chemerin released by adipocytes and albumen decreased in late pregnancy to accommodate the increased nutrition needs of the fetus, which might partially explain why GDM patients had lower chemerin levels in the third trimester [43].

Omentin may influence plasma glucose concentration by promoting insulin-stimulated glucose uptake in human subcutaneous and visceral adipocytes [15]. Pan et al. found that circulating omentin after fasting and 2 h post-glucose load were significantly decreased in patients with impaired glucose tolerance, and in those with newly diagnosed, untreated diabetes compared to healthy controls [44]. Similarly, El-Mesallamy et al. reported decreased circulating omentin levels in T2DM patients after adjusting for age or BMI [45]. The association of omentin and diabetes is not limited to patients with T2DM. Polkowska et al. revealed that circulating omentin concentration was lower in children with T1DM than in control children [46].

However, the data on omentin concentrations in GDM are limited. Our meta-analysis found that circulating omentin was lower in GDM patients than in controls. Interestingly, omentin is thought to be primarily expressed in visceral adipose tissue; however, in this analysis, omentin level was shown to be negatively associated with the amount of visceral adipose tissue. Visceral obesity has also been found to be associated with omentin secretion in adipose tissue. During the accretion of visceral fat, the release of free fatty acids and inflammatory cytokines into the portal vein increases, which leads to increased oxidative stress and IR. A decrease in omentin concentration, released from visceral adipose tissue might generate IR that causes GDM [32].

The subgroup analysis found that Caucasian, but not Asian or Australoid GDM patients had lower circulating omentin levels than controls. Circulating omentin levels were significantly higher in GDM patients younger than 30 years of age than in controls, and were significantly different in GDM patients with BMIs < 28 kg/m^2^ than in controls. Both age and BMI might influence circulating omentin concentrations in GDM patients. In homologous groups, omentin levels were higher in the second trimester (approximately 28 weeks gestation) than in the third trimester. The difference might have been the result of omentin production in both visceral adipose tissue and the placenta, which is the major source of omentin during pregnancy [32]. Thus, there will be increased omentin levels in early pregnancy, and omentin clearance during the later stages of pregnancy. Second, omentin may be increased in the second trimester of pregnancy because of increased fat accretion or decreased secretion from maternal adipose tissue [47–49].

Several study limitations should be taken into consideration. We excluded grey literature, such as conference abstracts and experimental animal studies, which may have led to selection bias. Heterogeneity may have influenced the interpretation of the results. There was substantial heterogeneity across studies, although random-effects models were applied to avoid statistical heterogeneity. Subgroup analysis of data stratified by seven potential sources also confirmed study heterogeneity (Table 2). However, sensitivity analysis and meta-regression analysis may have failed to identify all sources of heterogeneity because of insufficient data. Heterogeneity might also reflect differences in clinical variables, such as drug use, smoking status, alcohol consumption, exercise, environmental factors, diet adjustment, or an ideal index for reflecting obesity such as waist-hip ratio or waist circumference that were not considered. Considering the limited number of studies included in this meta-analysis, the relationship between circulating omentin levels and GDM requires further investigation.

## Conclusions

The results of this meta-analysis support omentin as a novel biomarker for the early diagnosis of GDM, which affects many pregnant women. Omentin deficiency may be involved in the pathogenesis of GDM. The decreased omentin levels in mothers with GDM, compared with healthy controls may result from impaired synthesis or release, but the mechanism for this requires further investigation. To date, the precise mechanisms by which omentin plays a role in glucose metabolism are not understood. In the future, we will carry out a large-scale prospective study to address the limitations.

## Supplementary information

**Additional file 1.** A full description of the search terms and strategy.

## Data Availability

All data generated or analyzed during this study are included in this article.

## Abbreviations

ACOG: American College of Obstetricians and Gynecologists
ADA: American diabetes association
ADIPS: Australasian Diabetes in Pregnancy Society
APJ: Apelin-angiotensin receptor-like 1
BMI: Body mass index
C&C: Carpenter and Couston
Cl: Confidence interval
ELISA: Enzyme linked immunosorbent assay
GDM: Gestational diabetes mellitus
IADPSG: International Association of Diabetes and Pregnancy Study Group
IR: Insulin resistance
NDDG: National Diabetes Date Group
NOS: Newcastle-Ottawa quality assessment scale
PRISMA: Preferred Reporting Items for Systematic Reviews and Meta-analysis
RARRES2: Retinoic acid receptor responder protein 2
SD: Standard deviation
SGLT-1: Sodium-dependent glucose transporter-1
SMD: Standardized mean difference
T1DM: Type 1 diabetes
T2DM: Type 2 diabetes
TIG2: Tazarotene-induced gene 2 protein
WHO: World Health Organization
