# Supplementary material for: Circulating apelin, chemerin and omentin levels in patients with gestational diabetes mellitus: a systematic review and meta-analysis
Source: Lipids Health Dis. 2020 Feb 22;19:26. doi: 10.1186/s12944-020-01209-7 (PMC7035755; doi:10.1186/s12944-020-01209-7)
Supplement: Supplementary file 1 — Additional file 1. A full description of the search terms and strategy. [file 12944_2020_1209_MOESM1_ESM.docx]

Search term for Pubmed inquiry

The PubMed search included (apelin [Mesh Terms] OR “apelin protein, human” [Text Word] OR “apelin, AGTRL1 ligand, human” [Text Word] OR “apelin-36 peptide, human” [Text Word] OR “apelin-31 peptide, human” [Text Word] OR “apelin-13 peptide, human” [Text Word] OR “apelin-28 peptide, human” [Text Word]) OR (chemerin [Mesh Terms] (OR “C-20 chemerin-derived peptide” [Text Word] OR chemerin proteins [Text Word] OR “proteins, chemerin” [Text Word] ) OR (omentin [Mesh Terms] OR “intelectin 1 (galactofuranose binding) protein, human” [Text Word] OR “omentin protein, human” [Text Word] OR “intelectin, human” [Text Word] OR “intestinal lactoferrin receptor, human” [Text Word] OR “hIntL protein, human” [Text Word] OR “intelectin 1, human” [Text Word] OR “omentin-1 protein, human” [Text Word] combined with (gestational diabetes mellitus [Mesh Terms] OR “diabetes, pregnancy-induced” [Text Word] OR “diabetes, pregnancy-induced” [Text Word] OR “pregnancy-induced diabetes” [Text Word]) OR “gestational diabetes” [Text Word] OR “diabetes mellitus, gestational” [Text Word].
